# Factors influencing color match of monochromatic resin composites: a narrative review

**DOI:** 10.3389/fdmed.2025.1742283

**Published:** 2026-01-12

**Authors:** Weibo Huang

**Affiliations:** School & Hospital of Stomatology, Wuhan University, Wuhan, China

**Keywords:** chameleon effect, color matching, dental restorations, mixing effect, monochromatic resin composites, optical properties

## Abstract

Monochromatic resin composite, a novel dental restorative material, is now increasingly utilized in dental restorations, especially for anterior teeth. Its benefits include appealing aesthetics and ease of handling. Achieving a natural tooth color match with monochromatic resin composites can be challenging, but recent advancements in color selection programs have significantly improved the accuracy and consistency of color matching in dental restorations. The paper aims to systematically analyze the key factors affecting the color matching of monochrome resin composite, including the optical properties of the material itself, clinical operation skills, external environmental factors and individual differences. Research indicates that the transparency of monochrome resin composites, light scattering characteristics, particle size distribution, and other factors all influence color matching accuracy. High-transparency resins are prone to color variations in low-light environments, while the wavelength, intensity, and curing duration of light sources during material hardening significantly affect color stability and final appearance. Additionally, clinical factors such as resin thickness and lamination techniques impact the final color presentation. External environmental factors-including oral lighting conditions and background color variations-also affect the color harmony between restorations and adjacent natural teeth. Unlike traditional layered resin composite, monochromatic resin composite exhibits color changes under illumination due to the “chameleon effect” and “mixing effect”. This simplifies the procedure and reduces the time required. They are particularly well-suited for correcting minor imperfections and restoring front teeth. However, monochromatic resin composites are not suitable for restorations requiring significant color variation. Therefore, alternative restorative techniques are utilized to improve color precision. The paper summarizes the factors affecting color matching of monochrome resin composite, which provides a theoretical basis for subsequent clinical practice and research. Meanwhile, with the development of material science and technology, the performance of monochrome resin composite in aesthetic restoration has been improved, so as to better meet the requirements of patients.

## 1 Introduction

Monochromatic resin composites are extensively utilized in dental restorations owing to their outstanding aesthetic effects and ease of manipulation, especially in the restoration of anterior teeth (1). In comparison with multicolored resin composites, monochromatic composites employ a single hue to simulate the natural color of teeth, posing a considerable challenge in color matching. Color matching serves as a crucial factor that influences the success of restorations. Ensuring that the match between the resin composite and the natural color of the patient's teeth is crucial for achieving an aesthetically pleasing outcome, directly influencing patient satisfaction and treatment results (2).

Multiple factors influence the color matching of monochromatic resin composites. Apart from the resin's inherent physical and chemical properties, clinical techniques and variations among individual patients also significantly contribute (3). The optical properties of the composite, especially its translucency, light scattering properties, and interaction with dental tissues are among the most direct factors influencing color matching. Existing studies indicate that resin composites with higher translucency are more challenging to blend with natural teeth, and the color difference becomes more pronounced under dim lighting conditions (4–6). Moreover, the interaction between the resin composite and the tooth is related not only to the material's optical properties but also the color matching of resin composites is influenced by factors including tooth position, cavity shape, cavity depth, and clinical handling during the restoration process. These factors affect the resin's performance under different lighting conditions, thereby increasing the risk of color mismatch. Therefore, to optimize the color matching of resin composites, it is essential to consider multiple factors to ensure a high degree of consistency with the natural tooth color.

In the process of color matching, clinical techniques play a crucial role and cannot be overlooked. The choice of curing the light source, along with its application method (such as curing time, light intensity, and wavelength), can impact the curing process of the resin and, as a result, the color of the resin (7). Over-curing may lead to a color shift, whereas under-curing can have a negative impact on the resin's physical properties and aesthetics (8). Moreover, the thickness and layering technique of the resin composite also affect the final color outcome. In complex restorations, improper handling can lead to obvious color discrepancies (9).

Individual patient differences also significantly impact color matching. Factors such as the natural color of the teeth, the structure of the tooth surface, and the lighting conditions in the oral cavity all affect the degree of color match between the resin and the teeth. These challenges render it challenging to attain ideal color matching using monochromatic resin composites, and this problem is even more evident in the aesthetic restoration of anterior teeth, which exhibit higher aesthetic requirements (10).

As resin composite materials continue to evolve, numerous novel resin composites have been developed with optimized optical and curing properties to enhance color matching precision. While a multitude of studies have investigated the factors influencing color matching in monochromatic resin composites, thorough reviews on these factors are still limited (11). Therefore, this review aims to conduct a systematic review of the existing literature and comprehensively analyze how the color matching of monochromatic resin composites is influenced by a variety of factors, including the optical properties of the material, clinical procedures, and patient-related variables. Key factors encompass the molecular structure and cross-linking density of the resin, which affect light absorption and reflection, as well as the type and concentration of colorants and additives used. Environmental conditions during processing, such as temperature and humidity, also play a role. Furthermore, clinical factors such as the dentist's color perception, the quality of the shade guide, and the lighting conditions during the selection process are critical for achieving accurate color matching. The goal is to provide solid scientific support for clinical applications and offer a reference for future research in related fields (12–14).

According to recent industry reports, the demand for dental aesthetics has experienced a substantial surge, with an annual growth rate of approximately 30% over the past decade, marking a 25-percentage-point increase from the previous rate of around 5% (15). This shift not only underscores the increasing demand for aesthetic restorations but also reflects society's growing focus on dental health and appearance. The advancements in dental restoration technology, especially in materials science and treatment techniques, have significantly contributed to the growth of the oral restoration market. According to industry reports, the global market for dental restoration and implant materials is projected to grow at a compound annual rate, reaching a substantial market size by 2029. Personalized services and technological innovations are key drivers for this growth, making oral restoration an integral part of modern healthcare (16).

## 2 Monochromatic resin composites and color matching phenomenon

### 2.1 Overview of the color matching phenomenon

The color matching phenomenon of monochromatic resin composites is grounded in unique physical and optical principles, encompassing structural color and the blending effect. Structural color arises from the incorporation of uniformly sized spherical nanofillers in dental resin composites, with diameters typically ranging from 100 to 500 nm (17), which are within or near the visible light wavelength spectrum. Upon entering the resin, light interacts with ordered nanoparticles, resulting in Rayleigh scattering when the particles are much smaller than the wavelength of light, and Mie scattering when the particles are comparable in size to the wavelength. This interaction also includes interference and diffraction effects. Light through precise control of the filler size and distribution, the material can selectively reflect specific wavelengths of light (typically within the red-yellow spectrum) while permitting other wavelengths to pass through.

**Figure 1:**
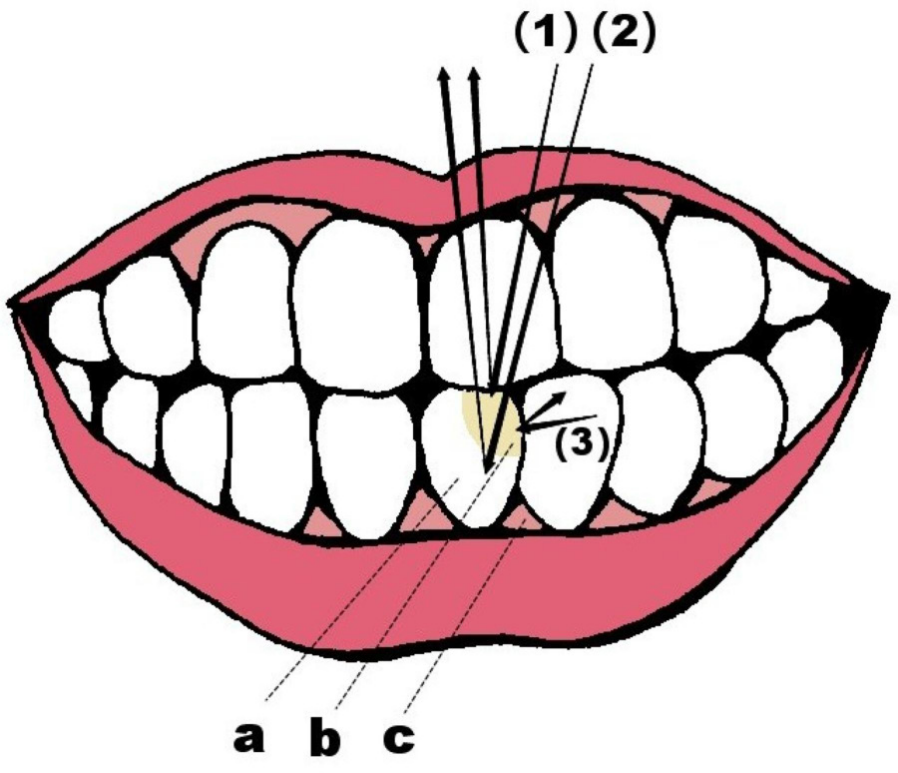
The mechanism of color formation in composite material **(a)** dental base, **(b)** monochromatic resin composite, **(c)** gingivae; (1) direct reflex, (2) reflection into the structure of the basal tooth affected by the composite material (main factor), (3) internal scattering of adjacent teeth.

The Blending Effect represents a distinctive characteristic of monochromatic resin composites in aesthetic restorations. The core mechanism, the key to color matching in these resins lies in their capacity to interact with ambient light, resulting in a tone that harmonizes with the surrounding environment. Monochromatic resin composites are capable of automatically adjusting their color and translucency in response to variations in external lighting conditions and the color of adjacent teeth. This allows the resin to blend seamlessly with the tooth, resulting in a more natural transition (18). This reflected light merges with the light reflected from the underlying dental tissues, as shown in Figure 1. Meanwhile, a small amount of light scattered by adjacent tooth structures conveys chromatic information, influencing the local color match in the restoration margin area (19). The final visual color results from the combined effect of the inherent color of the underlying tooth and the optical characteristics of the composite material. This optical integration effectively reduces the visual perception of chromatic differences, ensuring harmonious integration between the restoration and the surrounding dental arch. This effect is based on the microstructure and optical properties of the composite material, enabling the resin to match the tooth color and create a gradient effect between the surface and inner layers. This diminishes the perception of abrupt color changes, facilitating a more natural integration of the restoration with the surrounding teeth and preventing the formation of noticeable color blocks or unnatural boundaries between the restoration and natural teeth (20, 21). Furthermore, the resin can exhibit different hues based on the angle and lighting conditions, allowing the restoration to closely resemble natural teeth under various light sources (22). This enables the restoration to visually harmonize with the surrounding teeth, presenting a color that is consistent with their natural hue (23). Certain literature refers to this phenomenon as the “chameleon effect”, yet in the precise realm of color science, this term is not officially employed (24).

**Figure 2:**
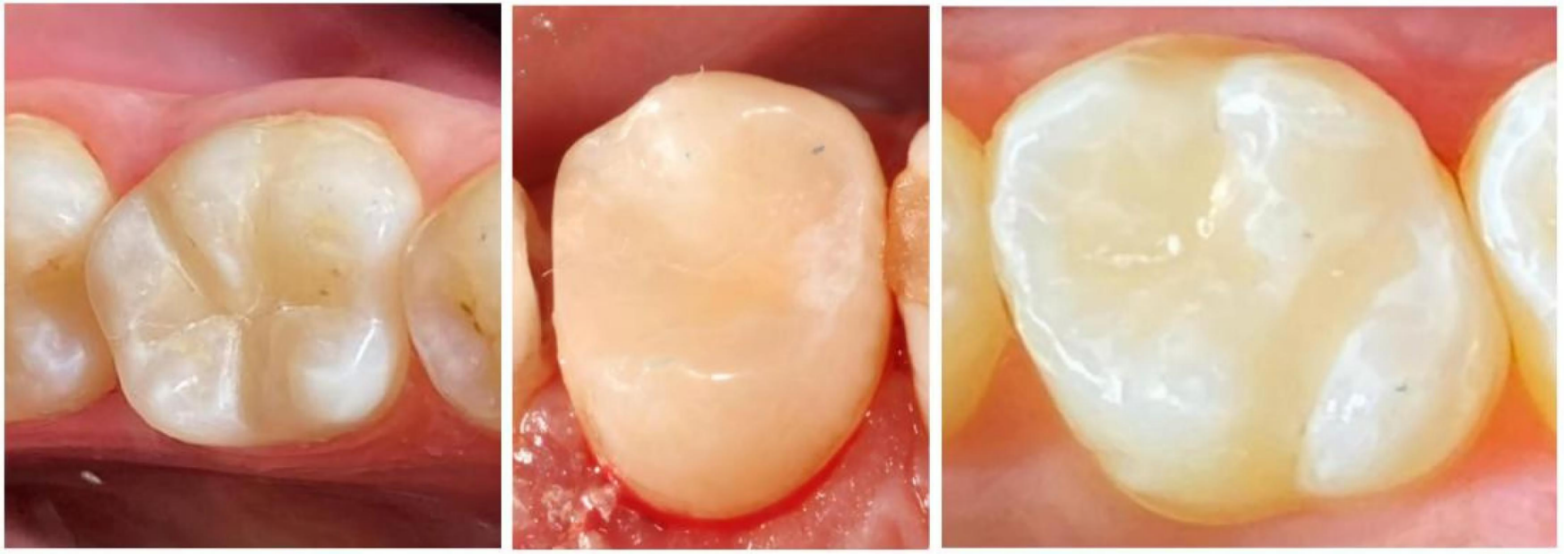
Showing the immediate clinical pictures of teeth restored with omnichroma [adapted from reference (16)].

Figure 2 illustrates the color-matching phenomenon of monochromatic resin composites, highlighting their role in achieving seamless restorations in modern dentistry. It shows the immediate clinical results of teeth restored with Omnichroma, reflecting how the material adapts to the natural tooth color. This supports the discussion on how monochromatic resin composites integrate with surrounding dental tissues to provide a natural, aesthetic outcome.

To objectively evaluate the effectiveness of color matching, dental research extensively employs the color space model developed by the International Commission on Illumination (CIE). This model characterizes color through three coordinates: L* denotes lightness (0 = black, 100 = white), a* signifies the red-green axis, and b* indicates the yellow-blue axis. The color difference between two colors is quantified by the Euclidean distance in the CIE Lab* space, represented as ΔE*ab, where a larger value indicates a greater perceived difference between the colors. The CIEDE2000 (Δ*E*00) formula was introduced to refine the weight parameters for a closer match to human visual perception, and it has become the gold standard for evaluating color differences in dental materials (25). This advancement was achieved through the work of the CIE's TC1-47 technical committee, which analyzed and tested existing color difference formulas and visual assessment data, leading to the development of CIEDE2000. The formula's precision and its ability to improve visual consistency in industrial color difference evaluation have been confirmed through multiple experimental data tests. The Perceptibility Threshold (PT) is defined as the critical value at which 50% of observers can detect a color difference, a concept that is crucial for understanding the uniformity of color perception and the development of color spaces. Multiple studies have shown that the typical threshold for color difference is set at Δ*E*00 ≤ 0.8, indicating that color differences below this value are generally imperceptible to the human eye. The Acceptability Threshold (AT) is defined as the critical value at which 50% of observers consider the color difference to be clinically acceptable, with a widely accepted threshold of Δ*E*00 ≤ 1.8. Research in color difference formulas such as CIEDE2000, CIELAB, and CIELUV has shown that these thresholds are crucial for ensuring color accuracy and consistency across various applications, from printing to color matching. Color differences exceeding this value may be considered unacceptable, and the restoration could be perceived as a failure by either the patient or the clinician (26).

### 2.2 Clinical application of monochromatic resin composites in different scenarios

Monochromatic resin composites are extensively utilized in dental restorations owing to their distinctive optical properties and ease of manipulation. Table 1 lists the monochromatic resin composite dental restorative materials commonly used in the market. They are especially common in anterior tooth restorations that have high aesthetic requirements and complex posterior tooth repairs (10). In anterior restorations, patients show a strong preference for a natural color match with the surrounding teeth. Monochromatic resin composites can adjust their color according to different lighting and environmental conditions, enabling them to achieve a seamless blend with the adjacent teeth. Moreover, these resins streamline the procedure and minimize the steps required for color adjustment, which is commonly necessary in traditional multi-layer composite restorations. Considering the variability in the color of anterior teeth and the complexity of oral lighting conditions, monochromatic resin composites are especially effective in achieving optimal restorative results (27–29).

**Table 1:** Common monochromatic resin composite dental restorative materials.

SN	Brand	Country	Filler type	Technical feature	Application Suggestions
1	Omnichroma	Japan	Nano-spherical filler(SiO_2_-ZrO_2_)	The “structural color” technology enables compatibility with 16 colors in the VITA Classic color series, covering all 16 shades from A1 to D4.	Direct filling of anterior and posterior teeth, especially for adjacent caries and large area defect.
2	Tetric-N-Ceram	Liechtenstein	Nano-hybrid filler	It has the characteristics of nano mixed filler, high x-ray blocking and “Chameleon Effect”.	Widely used in the clinical restoration of anterior and posterior teeth.
3	Ecosite One	Germany	Special polymer system	Based on the “Push-and-Flow” effect, it combines the easy operation of fluid with the high hardness of solidified material. The curing speed is fast, and the polishing and wear-resisting properties are excellent.	Designed for posterior bite.
4	Vittra APS	Brazil	Submicron mixing	Featuring APS (Advanced Polymerization System) technology, it exhibits exceptional “Chameleon Effect”-maintaining consistent transparency and seamless color blending before and after curing.	The treatment of shallow cavities and light-colored base color.
5	Aura Bulk	Australia	Glass filler	Bulk fill with monochromatic material allows deep cavity restoration without layered plasticization.	The posterior teeth were filled with large cavities.
6	Zenchroma	Germany	Bionic structure	With the “Chameleon Effect”, it maintains consistent transparency before and after curing, making it ideal for natural transitions in shallow cavities.	It is suitable for the anterior adjacent surface cavity and posterior cleft and fissure of the teeth with high aesthetic requirements.
7	Essentia Universal	USA	Micro-hybrid filler	The product demonstrates excellent surface gloss retention and achieves optimal performance when used with model liquid.	The high compressive strength makes it suitable for posterior tooth filling.
8	Harmonize	Australia	Nano-hybrid filler	Based on ART (Adaptive Response Technology) and the chameleon effect, the material provides low viscosity for easy manipulation during the initial shaping phase, while maintaining high integration with the surrounding dental structure before and after curing.	The restoration area has high requirements on aesthetics.

In comparison with traditional resin composite methods, monochromatic resin composites provide substantial benefits in clinical applications, particularly in reducing treatment duration and streamlining treatment procedures. The primary characteristic of these resins lies in their capacity to simplify the treatment process, rendering them especially appropriate for small-area defects or surface restorations, thereby markedly decreasing the number of steps and time needed for the restoration. Moreover, the optical properties of monochromatic resin composites, such as translucency, opalescence, and fluorescence, enable them to mimic natural teeth closely. In certain cases, this material can blend seamlessly with the patient's natural teeth, especially when dealing with teeth that have unique colors or minor defects. This helps prevent color mismatches, as the material's optical properties can adjust to the surrounding tooth color, resulting in an aesthetically pleasing outcome.

However, although monochromatic resin composites exhibit excellent adaptability in addressing minor color discrepancies and small defects, they do have limitations. In cases where there is a significant color difference between the teeth, these resins may not perfectly match the color of the adjacent teeth, as their color adaptability is limited by the underlying tooth color and the material properties. Under such circumstances, it may be necessary to combine resin composites of different shades or employ other auxiliary techniques (such as layered restorations) to further enhance the appearance of the restoration and ensure it harmonizes with the surrounding teeth. Furthermore, although monochromatic resin composites generally maintain good color stability under certain lighting conditions, slight color variations may still occur in special lighting environments. Therefore, in clinical practice, it is essential to select the appropriate materials and restoration strategies based on the patient's individual needs and oral environment.

**Figure 3:**
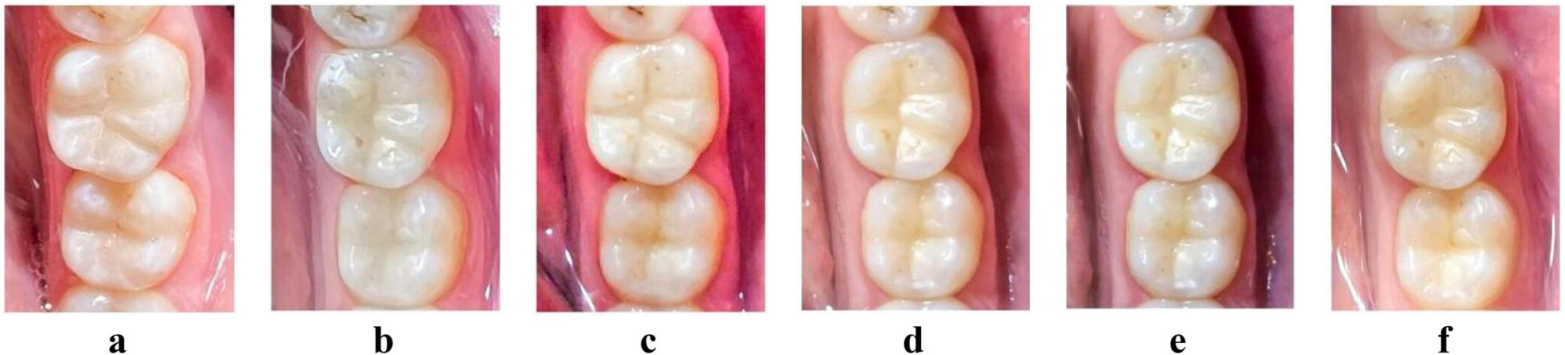
Application of monochromatic resin composites in different clinical scenarios [adapted from reference (20)].

Figure 3 shows photos of restorations on teeth 46 and 47 using Omnichroma and Tetric-N-Ceram resin composites (20). The images include follow-up photos taken before the restoration (a), 1 month (b), 3 months (c), 6 months (d), 9 months (e), and 12 months (f) after the restoration. These images not only showcase the restorative effects at various time points but also illustrate the color stability and adaptability of monochromatic resin composites over time. In dental restorations, the Omnichroma material exhibits excellent performance, featuring stable color fusion and minimal color discrepancies, guaranteeing a natural transition and an aesthetically pleasing outcome throughout the restoration process. In contrast, Tetric-N-Ceram demonstrates favorable initial outcomes; however, color deviations gradually emerge over time, and its color stability declines with extended use.

During the restoration of posterior teeth, where aesthetic demands are relatively low, monochromatic resin composites can effectively restore both the function and appearance of the teeth, particularly in cases with higher requirements for mechanical strength. As posterior teeth are generally less exposed to direct strong light, the requirement for color matching is less crucial, enabling monochromatic resin composites to maintain stable color and translucency. For most functional restorations of posterior teeth, monochromatic resin composites offer an efficient and cost-effective solution, avoiding complex procedures while restoring the occlusal function of these teeth.

In the restoration of large defects, the “blending” or “color-matching” effect of monochromatic resin composites enables the restoration to blend seamlessly with the surrounding tooth color and shape. Although restoring large defects poses considerable challenges and demands advanced technical expertise, monochromatic resin composites offer the flexibility to adapt to different thicknesses, fulfilling both aesthetic and functional needs. This proves particularly advantageous when repairing large defects in posterior teeth, as monochromatic resin composites not only produce excellent color fusion but also shorten the treatment time and reduce treatment costs for the patient (30, 31).

### 2.3 Comparison with traditional layered resin composites

Traditional layered resin composite restoration techniques provide significant aesthetic benefits by precisely layering materials of different hues to replicate the translucency, color gradient, and depth transitions present in natural tooth structures. However, this layered restoration technique is complicated, necessitating the dentist to possess extensive technical experience and advanced skills. Each layer of material requires precise control, and the restoration process generally takes longer, thereby increasing the overall treatment time and patient burden. Moreover, this technique necessitates a high degree of precision from the clinician at every step to guarantee a natural and long-lasting result (32).

In contrast, the advent of monochromatic resin composites has greatly simplified the restoration process. Thanks to their unique blending in effect, monochromatic resin composites are capable of automatically adjusting their hue in response to varying light conditions and the surrounding tooth color, thereby enabling them to blend more naturally with the patient's natural teeth. Owing to the optical properties of these materials, the restoration can harmonize seamlessly with the surrounding environment, which not only significantly reduces restoration time but also simplifies the procedure, especially in anterior restorations where rapid aesthetic results are highly sought after. Monochromatic resin composites are particularly well-suited for small defects and surface restorations, as evidenced by clinical studies showing a high success rate and patient satisfaction. Their aesthetic properties and low risk of leakage make them ideal for quicker and more convenient treatment options, as observed in anterior tooth aesthetic restorations.

However, despite their significant advantages in simplifying procedures and reducing treatment time, monochromatic resin composites, while offering benefits such as aesthetic appeal and ease of application, are not without their limitations, especially in complex dental restorations such as large defect repairs. They may find it challenging to achieve the same level of depth, layering effect, and fine detail. Layered resin composites offer anatomical restoration capabilities. For example, when restoring teeth with complex anatomical shapes, layered techniques can precisely replicate the tooth's three-dimensional structure and depth by applying resins with different shades and translucencies. In contrast, monochromatic resin composites may not attain the same level of intricate layering because of their single-hue nature. Therefore, for deep and complex tooth restorations, particularly when high demands are placed on the tooth's morphology, it may be necessary to combine other restorative techniques, such as layered restorations or the use of resin composites of different shades, to further enhance the aesthetic results and restoration quality.

Thus, although monochromatic resin composites offer distinct advantages in clinical practice, especially in simplifying operations and reducing treatment time, traditional layered resin composites still possess irreplaceable value in situations demanding high aesthetic standards and a sense of depth. When selecting clinical materials, dentists should take into account both the anatomical complexity and aesthetic requirements of the restoration site, carefully combining these two materials to achieve the best possible clinical results.

Monochromatic resin composites and traditional layered resin composites each possess distinct advantages and disadvantages. Monochromatic resin composites, owing to their distinctive optical characteristics, can automatically adjust to the surrounding tooth color changes, which simplify the restoration process and reduce treatment time. They are particularly well-suited for small-area defects and anterior restorations. Moreover, monochromatic resin composites excel in color matching, blending seamlessly with natural teeth. However, their drawback lies in the inability to achieve the same depth and layering effects as traditional layered resin composites, especially in large defects or complex restorations, where they may fall short in fine aesthetics. Additionally, under certain lighting conditions, the color stability of monochromatic resin composites may be affected, leading to slight color shifts.

In contrast, traditional layered resin composites, through the precise layering of colors, better simulate the natural translucency, color gradient, and depth transitions of teeth, making them ideal for anterior restorations where fine aesthetic details are required. Furthermore, layered resin composites exhibit superior color stability, as evidenced by studies that have shown minimal color alteration after exposure to staining agents and artificial aging, ensuring color consistency over an extended period. However, this technique is more intricate, necessitating longer restoration procedures and a higher level of expertise from the dentist, thereby increasing treatment duration and imposing greater requirements on patient care. Consequently, in clinical practice, the selection of materials should be determined according to the patient's restoration requirements, defect features, and the clinician's technical competence (33–35).

## 3 Factors affecting the color matching of monochromatic resin composites

### 3.1 Material properties

The effectiveness of color matching in monochromatic resin composites is largely influenced by the material's intrinsic properties, particularly its translucency and the size of the resin particles (36). Notably, translucency is a critical factor in the optical properties of resin composites, which is primarily determined by the compatibility between the refractive indices of the filler particles and the resin matrix. When light passes from the resin matrix into the filler particles, reflection and scattering occur at the interface between the two. The greater the difference in refractive indices, the more pronounced the light scattering effect, and the lower the translucency of the material (37). High-translucency resins are capable of mimicking the optical effects and color transitions of natural teeth due to their ability to transmit light effectively while scattering and refracting it in a controlled manner. However, achieving accurate color matching remains challenging due to the complexity of light interactions in these materials (35). Additionally, since underlying color tones are more readily exposed, this can impact the overall aesthetic outcome of the restoration (28). Conversely, resins with lower translucency might seem more noticeable under specific circumstances. Hence, managing translucency necessitates meticulous consideration in line with the restoration's demands, ensuring an optimal equilibrium between aesthetics and functionality (37).

The dimensions of the resin particles dictate their optical attributes, particularly their scattering and refractive properties, and play a crucial role. Resin composites play a crucial role in color matching by regulating light scattering and refraction through the distribution of fine particles, thereby influencing the color effect. Larger resin particles scatter light more extensively, resulting in a more uniform and smooth appearance. Smaller particles, in contrast, enhance the resin's optical translucency, with a microscopic structure closely resembling that of natural tooth tissue (38). However, particle size and distribution not only influence color matching but also influence the resin's mechanical properties and durability. For instance, resins with smaller particles generally exhibit better strength and wear resistance, yet may not attain the same natural optical effect as those with larger particles.

Resin composites are extensively employed in dental restorations; however, their long-term application is prone to staining and surface wear. Monochromatic resin composites are likely to discolor when exposed to common beverages like red wine, coffee, tea, carbonated drinks, and even saliva. Bleaching treatments are a common method for whitening teeth, but their effects on restorative materials have not been fully understood. Research has indicated that monochromatic resin composites demonstrate superior color stability and surface integrity post-bleaching compared to traditional resin composites, rendering them particularly suitable for patients seeking teeth whitening procedures (39).

### 3.2 External environmental factors

The color matching of monochromatic resin composites is not only determined by their intrinsic physical and chemical properties but is also significantly affected by external environmental factors. Factors such as background color, cavity size and shape, resin thickness, light curing conditions, external lighting, and tooth color all influence the color matching between the restoration and natural teeth. Therefore, clinicians must thoroughly consider these factors when performing resin composite restorations to ensure optimal outcomes (40).

The background color serves as a crucial factor that influences the color matching of monochromatic resin composites. The natural color of teeth is influenced by both the dentin and enamel. Dentin, which is typically yellow or gray, and enamel, which has a translucent quality with a bluish tint, together contribute to the overall color of teeth. Factors such as age, dietary habits, and oral hygiene can affect the appearance of teeth, often leading to a yellowing effect as enamel thins and dentin becomes more visible. These optical characteristics directly influence the performance of the resin when applied to different backgrounds. For instance, in patients with deep or yellowish dentin, a resin exhibiting higher translucency or specific tonal modifications is required to achieve a better color match. Studies have shown that both the translucency and color saturation of the resin composite affect its ability to match dentin. Highly translucent resins are better able to replicate the natural appearance of teeth, but in backgrounds with significant color differences, they may lead to discernible color differences between the restoration and adjacent teeth. This indicates that although monochromatic resin composites can effectively simulate the color of restored teeth, however, when significant color differences exist between teeth, the color of the restoration may not fully harmonize with adjacent teeth, thereby affecting the overall aesthetic outcome (41).

The size of the cavity also plays a crucial role in color matching; when the cavity is large, a thicker resin layer is required, and its color performance may vary accordingly. Resin thickness not only determines its final optical effect but also affects the color stability and depth. For smaller cavities, applying a thinner resin layer will make it more closely resemble the natural tooth color. Conversely, a thicker resin layer may lead to a color mismatch. Research indicates that once the resin layer surpasses a specific thickness, light transmission decreases, and the color performance may shift more towards the resin's intrinsic hue (34).

Resin thickness serves as an adjustable factor for color blending. The thickness of resin composite has a direct impact on its final color effect. Thinner resin layers blend more readily with the natural tooth color, whereas thicker layers may result in color distortion or an unnatural look. Thicker resin layers impact light transmission and the optical scattering properties of the resin, potentially leading to more pronounced color discrepancies in the restoration. Numerous studies have demonstrated a significant negative correlation between resin thickness and color matching accuracy (42).

Light curing plays a pivotal role in color matching (43). The curing process of resin composites directly impacts the final color stability and optical performance. Research has demonstrated that the wavelength of the light source has a substantial impact on the resin's final color. Various wavelengths of light can induce diverse color alterations in the resin during the curing process. Generally, longer wavelengths are inclined to generate warmer hues, whereas shorter wavelengths might lead to cooler shades. Excessive curing can result in color deviations, and thicker resin layers may cure unevenly owing to insufficient light penetration, thereby impacting the consistency and stability of the color. Conversely, incomplete curing could result in a reduction in the resin's hardness, adversely affecting its color and mechanical properties, which may cause discoloration or a decline in durability. Hence, precise control of light curing conditions, especially the wavelength of the light source and curing time, is vital for ensuring both color matching and long-term stability of resin composite restorations.

External lighting conditions are crucial for color matching and must not be ignored. The light source intensity, illumination angle, and the distance from the observer to the restoration all have a substantial impact on the resin composite color's appearance. Under varying lighting conditions, the resin color can change markedly, particularly when employing light sources with different color temperatures. Under a strong light source, the resin composite will exhibit a more saturated and deeper color; conversely, with a weaker light source, the resin will appear more transparent, potentially impeding the blending of the restoration with the surrounding tooth color. Furthermore, the distance between the observer and the restoration also influences color perception. Studies indicate that at close range, color details are more discernible, whereas at a greater distance, the restoration color tends to harmonize with the natural tooth color more closely.

In tooth restoration involving varying color layers, the incisal edge of natural teeth mainly consists of translucent enamel, whereas the cervical region is predominantly composed of more opaque dentin. Owing to their single tone and structural color features (like the “chameleon effect”), monochromatic resin composites might not fully replicate this multi-layered color variation. Research has shown that monochromatic resins exhibit poor performance in simulating the cervical region is closely related to the effect of the darker dentin background. For multi-colored teeth, such as those with translucent incisal edges, gray-toned middle sections, and yellow-toned cervical regions, the matching capability of monochromatic resins significantly decreases. Instrumental measurements show that the color differences (Δ*E* values) of monochromatic resins are typically higher than those of multi-colored resins on such multi-colored teeth. This indicates that when dealing with teeth that have uneven color distribution, monochromatic resins may not achieve ideal color consistency.

Generally speaking, color matching is easier on lighter backgrounds than on darker ones. On dark backgrounds, the color matching effect is less satisfactory. Thicker resin layers are more likely to exhibit larger color differences, whereas thinner layers lead to smaller color deviations. Under strong lighting conditions, color matching is better, while under weak lighting conditions, the results are less favorable. Smaller cavities are more conducive to achieving a natural color blend, while larger cavities may lead to color discrepancies. The wavelength of the curing light source, along with either excessive or insufficient curing time, can also adversely affect the color matching capability. Moreover, the color matching ability of monochromatic resin composites is further constrained by the complexity of the surrounding tooth color.

The background color serves as the optical foundation for the restoration, with its color characteristics “penetrating” through the translucent resin. The thickness of the resin serves as an adjustment factor, governing the extent to which the background color is obscured and the intensity of the resin's inherent color. Lighting conditions function as an objective criterion for assessing color matching, and non-standard lighting is one of the main factors leading to clinical color matching failures. The size of the cavity determines the degree to which the restoration can blend with the surrounding tooth structure through the “chameleon effect”. In clinical restorations, these factors must be thoroughly considered to attain the optimal color matching outcomes.

The disparities between anterior and posterior teeth carry significant implications for aesthetic restorations. In the anterior region, aesthetic requirements are more stringent, with a lower tolerance for color matching inconsistencies. Research indicates that monochromatic resin composites generally exhibit inferior color matching capabilities compared to multi-colored resins that are used in anterior restorations, particularly in complex areas that necessitate the simulation of the multi-layer structure of dentin and enamel. Multi-colored resins exhibit superior performance in common A2, B1, and B2 color shades, whereas monochromatic resins might not satisfy the stringent standards of translucency and multi-color demands in the anterior region. In contrast, the aesthetic requirements in the posterior region are relatively lower, and monochromatic resin composites generally perform better in fulfilling these needs. Clinical research suggests that for posterior restorations with mild to moderate aesthetic needs, multi-colored resins can achieve satisfactory aesthetic outcomes comparable to monochromatic resins.

### 3.3 Individual differences and social factors

The color-matching performance of monochromatic resin composites is markedly affected by the material properties and environmental factors, and individual differences along with social factors also exert a significant influence (38). People from different age groups, genders, and cultural backgrounds exhibit distinct aesthetic preferences. While these disparities do not directly affect the material's color matching, they can shape patients' expectations regarding the restorative outcome. For instance, elderly patients may place greater emphasis on the functionality and durability of the restoration, whereas younger patients may lean more towards achieving superior aesthetic outcomes. These disparities result in diverse expectations concerning the outcome of monochromatic resin composite restorations, which have become increasingly important. As society develops and aesthetic values shift, patients' demands for restorative outcomes have progressively risen, especially in their quest for “naturalness” and “invisibility”. This indicates higher expectations concerning the color, translucency, and seamless integration of restorative materials with natural teeth (44). Consequently, although monochromatic resin composites can fulfill certain restorative requirements, whether they can attain the ideal aesthetic effect depends on a comprehensive evaluation of the patient's individual needs and their definition of aesthetics.

Age exerts a considerable influence on the demands for aesthetic restoration. Generally, younger individuals tend to have higher expectations regarding the appearance of restorations, particularly in the case of anterior teeth restoration, where the color and shape are anticipated to closely mimic those of natural teeth. Conversely, older individuals may exhibit lower aesthetic demands; however, with the enhancement of living standards, numerous elderly patients are becoming increasingly concerned about the appearance of their dental restorations, preferring a natural and harmonious aesthetic. In such scenarios, monochromatic resin composites, with their exceptional adaptability and color-blending capabilities, can precisely fulfill the needs of these patients (45).

Gender also plays a role in influencing aesthetic demands. Men generally prioritize the functional and durable aspects of restorations, whereas women tend to place greater emphasis on details and aesthetics, especially in anterior restorations where the coordination of details and color harmony is crucial. Cultural background also significantly influences the aesthetic requirements for restorations (46). In certain cultures, white teeth symbolize health and beauty, whereas in others, the natural color and translucency of teeth are held in higher esteem. These cultural disparities require dental professionals to make adjustments during treatment to ensure that the restoration aligns with the patient's aesthetic expectations (47).

## 4 Conclusion and outlook

Monochromatic resin composites offer substantial advantages in fulfilling the aesthetic restoration requirements of contemporary individuals. They are capable of automatically adapting to the color of adjacent teeth, achieving a natural blending outcome, and are especially proficient in simulating the appearance of natural teeth in anterior restorations. The color - matching performance of monochromatic resin composites is affected by a variety of factors, including the optical characteristics of the resin materials, lighting conditions, resin thickness, as well as external environmental factors like the oral cavity's background and ambient lighting. All play crucial roles. To attain the best outcomes, a thorough consideration of both materials science and clinical techniques is essential. Clinicians need to have a comprehensive grasp of these influencing factors when employing monochromatic resin composites and should flexibly adapt their personalized techniques to achieve natural color harmony and aesthetic results.

With economic growth, there arises an increased demand for aesthetics. The demand for aesthetics is on the rise, and the application of monochromatic resin composites will become increasingly widespread. Patients' expectations for restorative outcomes are gradually shifting from focusing on functionality to emphasizing aesthetics, especially in anterior restorations and smile design. Owing to their efficient color-matching capability and ease of handling, monochromatic resin composites will be increasingly selected by both clinicians and patients. As materials science continues to advance, the utilization of light-cured resin composites in modern dental aesthetic restorations is on the rise, with color stability emerging as a key benchmark for evaluating treatment outcomes. Recent technological and material enhancements, particularly in the areas of optical property optimization, color stability, and long-term durability of monochromatic resin composites, are poised to mitigate existing constraints.

As the “beautiful smile” evolves into a personal asset with social and economic value, monochromatic resin composites are anticipated to be applied more extensively. As technology continues to advance and the demand for aesthetics rises, monochromatic resin composites are set to become the primary material for aesthetic restorations. In the future, monochromatic resin composites will play a more pivotal role in enhancing restoration quality, boosting treatment efficiency, and cutting costs, thereby propelling dental restorations into a new era.
